# Virus-Triggered Autoimmunity Was Associated With Hirschsprung's Disease Through Activation of Innate Immunity

**DOI:** 10.1155/2024/4838514

**Published:** 2024-10-26

**Authors:** Weiyong Zhong, Chaoting Lan, Yuqiong Chen, Kai Song, Zuyi Ma, Jixiao Zeng, Lihua Huang, Yan Zhang, Yun Zhu, Huimin Xia

**Affiliations:** Department of Pediatric Surgery, Guangdong Provincial Key Laboratory of Research in Structural Birth Defect Disease, Guangdong Provincial Children's Medical Research Center, Guangzhou Institute of Pediatrics, Guangzhou Women and Children's Medical Center, Guangzhou Medical University, Guangzhou 510623, Guangdong, China

**Keywords:** autoantibody, Hirschsprung's disease, innate immune, pathogen

## Abstract

**Background:** Hirschsprung's disease (HSCR) is a congenital enteric nervous system (ENS) disorder. Genetics cannot explain most sporadic cases. To explore the relationship between pathogen infection, autoantibodies, innate immune, and HSCR.

**Methods:** Pathogen microarray was conducted in the serum of the prospective neonatal abdominal distension (NAD) cohort, consisting of 56 children followed for at least 6 months until the final diagnosis of HSCR was determined or excluded. We conducted an autoantibody microarray in an HSCR cohort, which is comprised of diagnosed HSCR patients (HSCR) and healthy control subjects (HC). RNA-seq of colon tissues from aganglionic and ganglionic segments of HSCR patients was performed.

**Results:** Experimental results show that the serum lgM and lgG of enterovirus 71 (EV71) were significantly higher in HSCR than in the gastrointestinal dysfunction (GI) group, with a prediagnose value reaching area under the curve (AUC) over 0.76. We discovered that a group of autoantibodies were significantly higher in HSCR including neuronal pentraxin 1 (NPTX1), amyloid, neuron lysate, and myelin-associated oligodendrocytic basic protein (MOBP) than that in the HC group. These four autoantibodies could distinguish HSCR from the HC group, with a combined AUC of over 0.90 using both serum IgG and IgM. Further analysis showed that wide activation of innate immune pathways, including toll-like receptor (TLR) signaling pathway, neutrophil-to-lymphocyte ratio (NLR) signaling pathway, red cell distribution width to lymphocyte ratio (RLR) signaling pathway, and cyclic adenosine monophosphate (cAMP) signaling pathway in aganglionic compared to ganglionic segments of HSCR.

**Conclusion:** This study suggested that virus-triggered autoimmunity may contribute to HSCR through activation of innate immunity, which facilitates the diagnosis and prevention of HSCR.

**Summary**

• The study reveals that virus-triggered autoimmunity may contribute to Hirschsprung's disease (HSCR) through the activation of innate immune pathways.
• This finding enhances our understanding of the relationship between HSCR, pathogen infection, autoantibodies, and innate immunity, offering potential improvements in diagnosis and prevention strategies for HSCR.

## 1. Introduction

The Hirschsprung's disease (HSCR) is a congenital birth abnormality characterized by a lack of ganglion cells (aganglionosis) in the distal colon. Although genetic mutations account for roughly 50% of familial cases of HSCR, the cause of most sporadic cases remains unknown [1]. In previous reports, the intestinal microbiota is influenced by ingested food and the local environment, which in turn influences the enteric nervous system (ENS) [2]. A lack of microbiota in the gut modifies the number of neurons in the enteric ganglion [3]. Induction of dysbiosis by broad-spectrum antibiotics reduces neuronal density and accelerates the onset of acute constipation in a mouse model of HSCR [4]. Numerous metabolites produced by bacteria can directly or indirectly affect the ENS and central nervous system (CNS). It has been documented that gut microbes are altered in neurodevelopmental disorders such as HSCR, autism, and psychiatric disorders, as well as neurodegenerative disorders such as Parkinson's disease or Alzheimer's disease [2]. Accumulating evidence suggests that viral, parasitic, and bacterial infections affect the ENS, increasing gastrointestinal motility and dysfunction [5, 6]. However, whether environmental factors contribute to increased susceptibility to HSCR remains unclear.

Virus infection could trigger an autoimmune response and activation of the innate immune system. Viruses are one of the pathogenesis of various autoimmune diseases through several mechanisms such as molecular mimicry [7]. Among these viruses, enteric viruses, cytomegalovirus (CMV), and Epstein-Barr viruses (EBV) serve as good examples to induce the initiation and development of autoimmune diseases [8]. Autoantibodies increase in these viral diseases and lead to cytotoxicity, phagocytosis cell surface receptor binding, and immune complex-mediated damage [9]. Innate immunity mediates or protects from these pathogenic effects through pattern recognition receptors (PRRs) [10] and results in the production of type I interferon (IFN-I), cellular alarm signals (interferons), and a range of cytokines and chemokines that coordinate immune responses [11]. In systemic lupus erythematosus (SLE), toll-like receptor (TLR)7 and TLR9 could sense immune complexes derived from autoantibodies and self-nuclear antigens to modulate adaptive immune response [12].

In this study, we found that increased enterovirus 71 (EV71) antibodies in patients with neonatal abdominal distension (NAD) are associated with HSCR. We also observed the diversity of autoantibodies was higher in HSCR patients than in healthy control (HC). These antibodies could diagnose HSCR in the period of the neonate. In colon tissues from aganglionic and ganglionic segments of HSCR patients, wide activation of innate immune pathways in aganglionic compared to ganglionic segments of HSCR was discovered. These results suggest that pathogen infections may contribute to HSCR through the activation of autoimmunity, which may facilitate the diagnosis and prevention of HSCR.

## 2. Materials and Methods

### 2.1. Cohorts Study

Both HSCR (HSCR = 45; non-HSCR = 5) and newborn abdominal distention (HSCR = 18; non-HSCR = 38) cohorts were enrolled in 2019 and 2020. The neonatal abdominal distention cohort comprised patients who presented to the neonatal intensive care unit (NICU) with ambiguous newborn bloating and a possible clinical result of HSCR. Participants in the cohort were contacted by phone every 6 months. Additionally, the cohort's serum was enlisted to test for IgM and IgG autoantibodies.  Table 1 provides information about the study cohorts.

### 2.2. Preparation of Serum

Three milliliters of peripheral blood were collected in 4 mL vacuum tubes without additives from fasting subjects for routine examinations for HSCR, infant abdominal distention groups, or physical examinations (control). The collected blood was processed within 2 h. After centrifugation at 4°C and 1500 rpm for 20 min, the serum was obtained. Further centrifugation at 4°C and 3000 rpm for 15 min was carried out. Residual serum was applied for this study and stored at −80°C.

### 2.3. Pathogen and Autoantibody Microarray

Pathogen (Cat. no. PA010) with 120 proteins and autoantibody microarray (Cat. no. PA001) with 114 proteins provided by GeneCopoeia (Rockville, MD, USA; Supporting Information 1: Table S1 and Supporting Information 2: Table S2). The serum screening was performed according to the manufacturer's instructions. The experimental protocol and data processing are provided by GeneCopoeia.

The serum was treated with Dnase-I and then diluted 1:50 in PBST buffer. Mixed buffer as a negative control. The diluted serum sample is incubated with the protein array, binding of antibodies to array proteins was quantified using Cy3-coupled anti-human IgG (1:1000) and Cy5-coupled anti-human IgM (1:2000) antibodies and a Genepix 4200A scanner (molecular device) equipped with 532 and 635 nm lasers. The net fluorescence intensity (NFI) and signal-to-noise ratio (SNR) of each array protein were calculated using GenePix Pro software (V.7, molecular devices) to determine the signal strength of IgG and IgM. The data were standardized using robust linear modeling (RLM) techniques incorporating internal positive controls to produce an antibody score (ABS), which is a quantifiable measure of each antibody's ability to bind to a matching antigen.

### 2.4. RNAseq Performance and Analysis

We processed colon tissues from aganglionic and ganglionic segments of HSCR patients using the RNAeasy kit (Qiagen, 74106) to remove ribosomal RNA and isolate total RNA. The NEBNext Ultra Directional RNA Library Prep Kit for Illumina (#E7420, NEB) was used to prepare the library, and sequencing was performed on the Illumina Hiseq X Ten platform with a paired-end module of 150 bp. Novogene was responsible for library preparation, and Trimmomatic (v0.39) was employed to eliminate adapter sequences from raw reads before aligning them to UCSC hg19 using STAR (v2.7.0d). Differential express genes (DEGs) were analyzed using limma (*R* package) with *q* < 0.05.

### 2.5. Receiver Operating Characteristic (ROC) Curve for Diagnose Performance

To evaluate the diagnostic utility of autoantibodies, we constructed a ROC curve, derived a cutoff value, and calculated the area under the curve (AUC). The sensitivity and specificity of the markers were measured to determine the diagnostic precision. The multiple indexes were combined as logit (*p*) using logistic regression for the evaluation of combined diagnosis performance.

### 2.6. Statistical Analysis

*R* and GraphPad Prism 9.0 (GraphPad Software Inc., CA, USA) were used for the statistical analysis of the data. We analyzed the quantitative variables using the *t* test. All *p* values are from two-tailed testing, and significance was defined as a *p* value less than 0.05.

## 3. Results

### 3.1. Analysis of Pathogen Microarrays

**Figure 1:**
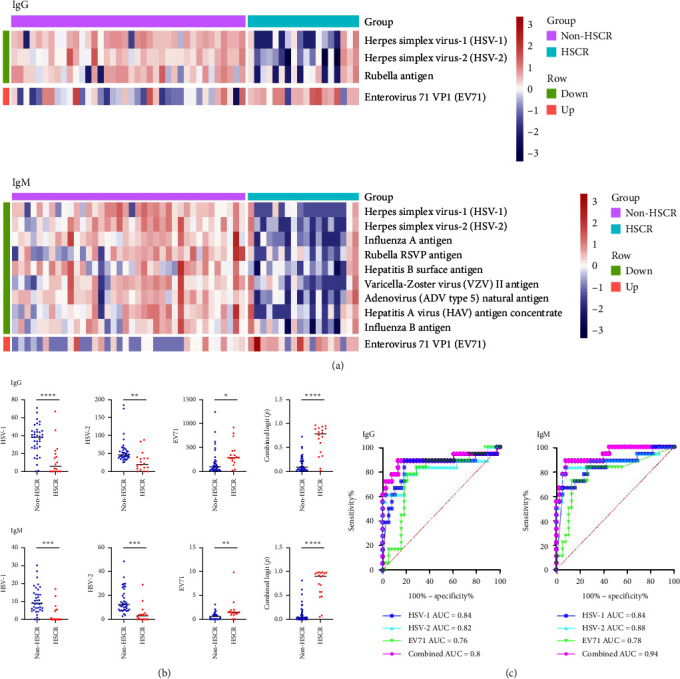
Antigen microarray in serum from NAD cohort exploring EV71 infection associated with HSCR. (A) The heatmap shows the average normalized fluorescent intensity of significantly altered autoantibodies in the serum of 18 patients with HSCR and 38 non-HSCR patients (*p*  < 0.05). The top section of the heatmap represents human IgG autoantibodies, while the bottom section represents human IgM autoantibodies. (B) The scatter plots compare the level of pathogen antibodies (HSV-1, HSV-2, and EV71), and the combined index of them shown as logit (*p*) using logistic regression in patients with HSCR than in healthy control subjects. *p* Values from Student's *t* test. *⁣*^*∗∗∗∗*^*p*  < 0.0001, *⁣*^*∗∗∗*^*p*  < 0.001, *⁣*^*∗∗*^*p*  < 0.01, *⁣*^*∗*^*p*  < 0.05. (C) ROC curve of individual and combined pathogen antibodies (HSV-1, HSV-2, and EV71) for HSCR diagnosis. EV71, enterovirus 71; HSCR, Hirschsprung's disease; NAD, neonatal abdominal distension; ROC, receiver-operating characteristic.

To explore whether pathogen infection was associated with HSCR pathogenesis, pathogen microarray detection was conducted on the serum of 56 children in the prospective NAD cohort, which were followed for at least 6 months until the final diagnosis of HSCR was determined or excluded. We found both lgG and lgM of EV71 were significantly higher in HSCR than non-HSCR NAD patients, while herpes simplex virus (HSV)-1 and HSV-2 lgM and lgG were higher in non-HSCR NAD patients (Figure 1A,B). The diagnosis performance of HSCR and non-HSCR with AUC using lgG of HSV-1, HSV-2, and EV71 were 0.84, 0.82, 0.76, AUC using lgM were 0.84, 0.88, 0.78 for AUC using lgM, and the combined AUC of 0.89 (a sensitivity of 88.89% and a specificity of 86.84%, cutoff : 0.31) and 0.94 (a sensitivity of 88.89% and a specificity of 89.84%, cutoff : 0.42) (Figure 1C). Our findings suggest that the activation of retrotransposons for autoimmunity in HSCR may be due to pathogenic infection, which could facilitate the early diagnosis of HSCR.

### 3.2. Analysis of Autoantigen Microarrays

**Figure 2:**
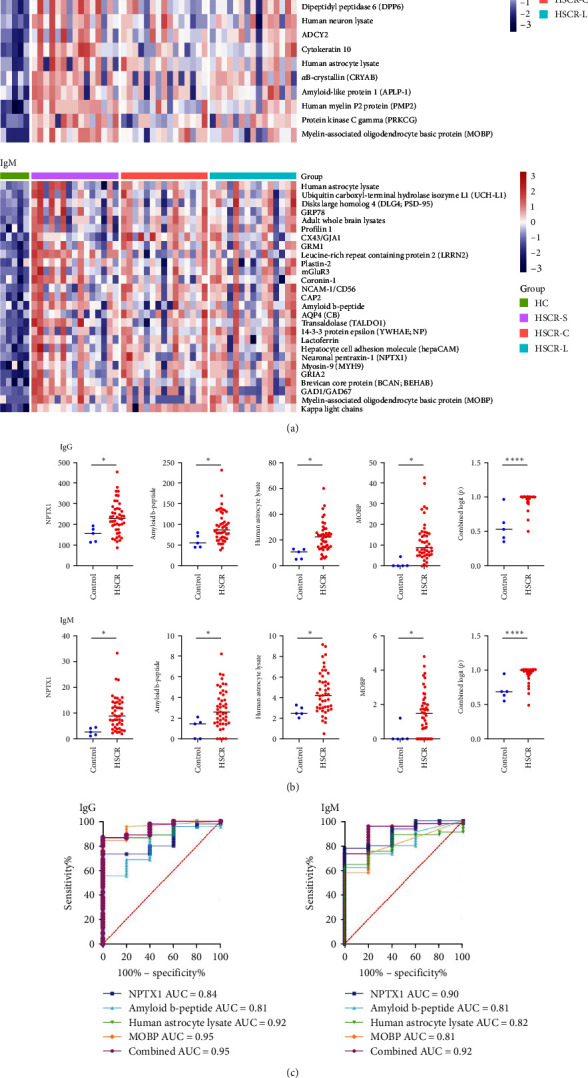
Autoantigen microarray in serum from HSCR cohort showing increased autoantibodies in HSCR patients. (A) The heatmap displays the average normalized fluorescent intensity of significantly upregulated in serum from 45 patients with HSCR and five healthy subjects (*p* < 0.05). Human IgG and IgM autoantibodies are shown in the top and bottom halves, respectively. The heatmap was generated based on group averages using Euclidean distance metric. (B) Scattered plots compare the level of each autoantibody (NPTX1, amyloid, neuron lysate, MOBP), and the combined index of these four autoantibodies shown as logit (*p*) using logistic regression in patients with HSCR than in healthy control subjects. *p* Values from Student's *t* test. *⁣*^*∗∗∗∗*^*p* < 0.0001, *⁣*^*∗*^*p* < 0.05. (C) ROC curve of individual and combined autoantibodies (NPTX1, amyloid, neuron lysate, and MOBP) for HSCR diagnosis. HSCR, Hirschsprung's disease; ROC, receiver-operating characteristic.

To verify whether autoantibodies were produced in Hirschsprung, we performed autoantigen microarray on the serum of 45 children with HSCR including three subtypes of HSCR (C-HSCR, S-HSCR, and L-HSCR) and five HCs. It was shown that a large group of upregulated autoantibodies in HSCR patients compared with HCs in both IgG-type and IgM-type microarrays (Figure 2A). Specifically, neuronal pentraxin 1 (NPTX1), amyloid, neuron lysate, and myelin-associated oligodendrocytic basic protein (MOBP) are significantly higher in HSCR patients compared to healthy subjects (*p*  < 0.05; Figure 2B). These autoantibodies could distinguish HSCR patients and HCs with AUC reaching at 0.84, 0.81, 0.92, and 0.95 in the IgG-type, 0.90, 0.81, 0.82, and 0.81 in the IgM subtypes, and the combined AUC at 0.95 (a sensitivity of 80% and a specificity of 100%, cutoff : 0.96) and 0.92 (a sensitivity of 95.56% and a specificity of 80%, cutoff : 0.70; Figure 2C). These results suggested that autoantibodies significantly increased in HSCR, which could be applied in diagnosis of HSCR.

### 3.3. Activation of Innate Immune in HSCR

**Figure 3:**
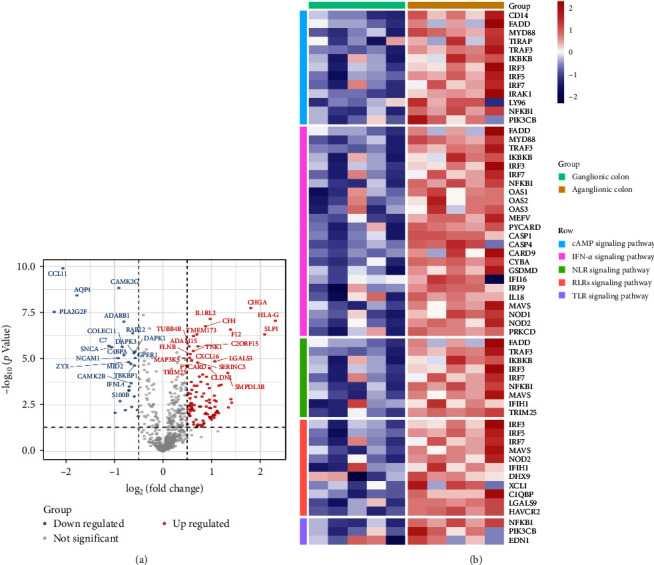
RNAseq analysis from the aganglionic versus ganglionic segments of HSCR patients reveals activation of innate immune pathways. (A) The volcano plot shows the DEGs between paired aganglionic versus ganglionic segments of HSCR patients (*N* = 5), with fold change (log_2_) and adjusted *p* value (–log_10_) indicated. (B) The heatmap plot reveals the upregulated genes in innate immune pathways in the aganglionic segments of HSCR patients compared to ganglionic segments. DEGs, differentially expressed genes; HSCR, Hirschsprung's disease.

As virus infection could trigger autoantibodies and activate the innate immune system, RNAseq analysis of colon biopsies from five HSCR patients was carried out to explore whether activation of the innate immune was implicated in the pathogenesis of HSCR. We compared the gene expression profiles of aganglionic segments to those of ganglionic segments in HSCR patients and identified 297 significantly differentially expressed genes (DEGs) among 721 genes related to innate immunity, with 211 genes upregulated (e.g., ACTG1, NOD2, LY96, and POLR3H) and 86 genes downregulated (e.g., GBP2, C4BPA, EIF2AK2, and RIPK2; Figure 3A). A comprehensive analysis of 44 pathways revealed the activation of innate immune-related signaling pathways, including TLR signaling pathway, neutrophil-to-lymphocyte ratio (NLR) signaling pathway, red cell distribution width to lymphocyte ratio (RLR) signaling pathway, and cyclic adenosine monophosphate (cAMP) signaling pathway in aganglionic compared to ganglionic segments of HSCR. The heatmap reveals that the majority of genes, including cluster of differentiation 14 (CD14), Fas-associated with death domain protein (FADD), myeloid differential protein-88 (MYD88), and inhibitor of kappa-light polypeptide gene enhancer in b-cells kinase beta (IKBKB), were upregulated in the aganglionic segments of HSCR patients compared to ganglionic segments. (*p*  < 0.01Figure 3B). These results suggest that the innate immune pathways were activated in HSCR.

## 4. Discussion

HSCR is a congenital ENS disorder. This study suggested that pathogen infection might trigger autoimmune in HSCR. We discovered that innate immune-related pathways are broadly activated in aganglionic segments relative to the ganglionic segments of HSCR patients. In previous studies, viral infection, genotoxic stress, and physiological changes have been shown to activate innate immune pathways, which are associated with neurological diseases. The level of pathogens in the serum of patients with HSCR in the early stage, whereas the level of autoantibodies increases in the serum in the later stage.

In this study, we also focused on the prediagnosis and diagnosis of HSCR and developed a noninvasive, stable, efficient, and sensitive microarray-based diagnostic technique. We were able to distinguish HSCR patients early in the neonatal period by combining lgM/lgG of HSV-1, HSV-2, and EV71, with AUC over 0.90. Additionally, we observed distinct differences and persistent trends in IgG and IgM microarray in HSCR cohorts, as evidenced by NPTX1, amyloid, neuron lysate, and MOBP, with a value reaching an AUC over 0.90.

Currently, the primary treatment for HSCR is a surgical intervention to remove functional blockage caused by a deficiency of ganglion cells, which extends and enhances the life of HSCR patients [13, 14]. However, after surgery, many children with HSCR remain asymptomatic, yet enterocolitis is still observed in 35% of cases [15–17]. Additional surgical complications may include stool leakage, anastomotic stenosis, anastomotic leakage with abscess, and chronic constipation. In our study, we observed a significant association between HSCR and elevated levels of IgM and IgG against EV71 in the serum of HSCR patients compared to those with gastrointestinal dysfunction (GI). These findings suggest a potential role for viral infection in the pathogenesis of HSCR. Patients infected with EV71 often present with elevated levels of cytokines [18–21]. Cytokines serve as terminal effector molecules in the innate immune response against viruses and play crucial roles in viral clearance. Studies have indicated that TLR7 and TLR8 may be involved in recognizing EV71 genomic RNA [22]. Knockout mice lacking the TLR9 gene exhibit significant lesions in the brain, small intestine, and forelimbs after EV71 infection, suggesting that TLR9 mediates protective immune responses against EV71 infection in mice [23]. Conversely, overexpression of melanoma differentiation-associated (MDA)5 and Retinoic acid-inducible gene I (RIG-I) enhances the type I interferon response induced by EV71 genomic RNA, indicating potential roles for MDA5 and RIG-I in mediating the innate immune response against EV71 infection [24]. The TLR3 signaling pathway in macrophages is indispensable for invariant natural killer T cells (iNKT) cell-mediated protective immune responses against EV71 infection [25].

In the case of EV71 infection, it is possible that the virus triggers an immune response, including the production of autoantibodies, which then cross-react with antigens in the ENS. This cross-reactivity may result in inflammation and damage to the ENS, thereby promoting the development of HSCR. Additionally, our literature review and the elevated levels of autoantibodies observed in HSCR patients further support the concept of autoimmune involvement in the disease. Specifically, the identification of autoantibodies targeting proteins such as NPTX1, amyloid, neuron lysate, and MOBP suggests that these proteins may play a role in the pathogenesis of HSCR through molecular mimicry mechanisms initiated by viral infection. Moreover, our RNAseq analysis revealed widespread activation of innate immune pathways in the aganglionic segments of HSCR patients compared to ganglionic segments. This observation indicates that viral infection may not only trigger autoimmune responses but also activate innate immune pathways in affected colon tissue, leading to inflammation and dysregulation of gut motility. In conclusion, our results support the hypothesis that virus-triggered autoimmune responses, possibly through molecular mimicry mechanisms, play a role in the pathogenesis of HSCR. The activation of innate immune pathways in affected colon tissue further highlights the complex interplay between viral infection, autoimmunity, and innate immunity in the development of this congenital disorder. These findings provide valuable insights into potential diagnostic and preventive strategies for HSCR.

Experimental models have demonstrated that mycophenolate mofetil, statins, artemisinin, high-dose ibuprofen, and vitamin A deficiency can inhibit the colonization of ENS precursors in the intestinal system. This suggests that any condition that impedes fetal cell proliferation during the critical period of ENS precursor migration increases the risk of HSCR. These conditions, such as antimitotic drugs, smoking, cocaine, alcohol, placental insufficiency, hypertension, folate deficiency, and other causes, have been identified as contributing factors to intrauterine growth restriction [26–30]. Therefore, taking a daily multivitamin before and during pregnancy, using pharmaceuticals judiciously, maintaining a healthy weight, abstaining from alcohol and drugs, and avoiding identified risk factors may help to prevent HSCR. Our study suggests that preventing pathogen infection can help to prevent the occurrence of HSCR. In children with pathogen infections such as EV71, abdominal distension is often associated with subsequent HSCR. This suggests that preventing pathogen infection may be a viable approach to preventing HSCR. Additionally, pathogen microarrays for abdominal distention screening in children may enable early detection of potential HSCR patients, thus facilitating early intervention.

This research is limited by the lack of experimental confirmation of the causal link between innate immunity, pathogen infection, and the development of HSCR. Further investigations can be conducted using animal models and examining the cellular level. By employing sequencing technologies, specific genes affected by transposon activation can be identified and studied for gene-specific precision therapy. In conclusion, our findings suggest that the activation of innate immune pathways from pathogen infection may contribute to HSCR, with the production of autoantibodies. This discovery can facilitate early detection, treatment, prevention, and management of HSCR.

## Figures and Tables

**Table 1:** Sample information of HSCR and NAD cohorts.

Characteristic		HSCR (*N* = 50)			NAD (*N* = 56)		
		HC (*N* = 5)	HSCR (*N* = 45)	*p*	non-HSCR (*N* = 38)	HSCR (*N* = 18)	*p*
Gender							
Female		0 (0%)	6 (13.3%)	1.000	14 (36.8%)	6 (33.3%)	1.000
Male		5 (100%)	39 (86.7%)	—	24 (63.2%)	12 (66.7%)	—
Age (left: month and right: day)	Median (IQR)	4.0 (3.0–5.0)	6.0 (4.0–9.0)	0.056	29.0 (15.0–42.0)	23.5 (8.0–29.0)	0.176
Gestational age (weeks)	—				37.4 ± 0.53	37.8 ± 0.6	0.5791
Birth weight (g)					3031 ± 91.1	3168 ± 115.2	0.3561
Vaginal birth (%)					51.9	52.9	0.9455

*Note:* Two-tailed *χ*^2^ test. The data were expressed as $\overset{-}{X}$ ± s when not specified.

Abbreviations: HC, healthy control; HSCR, Hirschsprung's disease; IQR, interquartile range; NAD, neonatal abdominal distention.

## Data Availability

The data underlying this article will be shared on reasonable request to the corresponding author.

## Supporting Information

Supporting Information 1 Table S1. Customized list of pathogens.

Supporting Information 2 Table S2. Customized list of autoantibodies.
