# Supplementary material for: Virus-Triggered Autoimmunity Was Associated With Hirschsprung's Disease Through Activation of Innate Immunity
Source: J Immunol Res. 2024 Oct 26;2024:4838514. doi: 10.1155/2024/4838514 (PMC11531361; doi:10.1155/2024/4838514)
Supplement: Supporting Information 1 — Table S1. Customized list of pathogens. [file 4838514.f1.docx]

**Supplementary table 1.**

| **Customized list of pathogens** | | | | | |
| --- | --- | --- | --- | --- | --- |
| **#** | Pathogen ID | **#** | Pathogen ID | **#** | Pathogen ID |
| 1 | Histone (H1/H2A/H2B/H3/H4 mix) | 41 | Glutamate decarboxylase 2 (GAD2/GAD65) | 81 | Norovirus vp1 |
| 2 | AChR3 (muscle) | 42 | GPIb/GPIIbIIIa/GPIV | 82 | Nucleosome |
| 3 | Adenovirus (ADV type 5) natural antigen | 43 | H1N1 HA (?TM) (aa18-529, Strain A/Michigan/45/2015) | 83 | Ovalbumin (OVA) |
| 4 | Alpha enolase | 44 | Haemophilus influenza OmpP2 | 84 | Parvovirus VP1 (aa 1-781) |
| 5 | Alpha Fodrin | 45 | HCoV-229E Spike S1/2 | 85 | Proteolipid protein(PLP) |
| 6 | Aquaporin 4 (AQP4) | 46 | HCoV-HKU1 Spike S1 | 86 | Pseudomonas fluorescense I2 |
| 7 | Arrestin, Beta 1/(Human ARRB1) | 47 | HCoV-HKU1 Spike S1/2 | 87 | Respiratory Syncytial Virus (RSV) long strain |
| 8 | B cell activating factor (BAFF) | 48 | HCoV-NL63 S/40604 | 88 | Ro/SSA (52kd&60kd mix) |
| 9 | Beef_Bos taurus | 49 | HCoV-OC43 Spike S1/2 | 89 | ROTAVIRUS SA-11 ANTIGEN |
| 10 | Bermuda_Cynodon dactylon | 50 | Hepatitis A Virus (HAV) Antigen | 90 | RUBELLA GRADE IV ANTIGEN PBS |
| 11 | CANDIDA ALBICANS | 51 | Hepatitis A Virus (HAV) Antigen, concentrate | 91 | Rubella RSVP Antigen |
| 12 | Carboxypeptidase H | 52 | Hepatitis B surface antigen | 92 | RUBELLA VIRUS GRADE III ANTIGEN |
| 13 | Casein (milk protein) | 53 | Herpes simplex virus-1 (HSV-1) | 93 | RUBEOLA ANTIGEN |
| 14 | Cat dander | 54 | Herpes simplex virus-2 (HSV-2) | 94 | SARS-CoV-1 NP Protein |
| 15 | Cedar,Red_Juniper rus virginiana | 55 | Honey Bee_Apis mellifera | 95 | SARS-CoV-1 Spike S1 |
| 16 | CENP-A/CENP-B (mix) | 56 | House Dust | 96 | SARS-CoV-1 Spike S1 RBD |
| 17 | Chitinase 3-Like 1 (Cartilage Glycoprotein-39) | 57 | Inactivated Respiratory Syncytial Virus Antigen | 97 | SARS-CoV-2 NCP 094 |
| 18 | Cholinergic Receptor Muscarinic 3 (CHRM3) | 58 | Influenza A antigen | 98 | SARS-CoV-2 NCP 40588 |
| 19 | CMV Ext-2, enriched for cell surface glycoprotein antigens | 59 | Influenza B antigen | 99 | SARS-CoV-2 NCP (40588-V07E) |
| 20 | CMV III Antigen, enriched for pp65 | 60 | Jo-1 | 100 | SARS-CoV-2 Spike S1 40591 |
| 21 | CMV-G, whole cell extract | 61 | KU-P70/P80 | 101 | SARS-CoV-2 Spike S1 C091 |
| 22 | CMV-M Concentrate, nuclear extract and ER antigens | 62 | La/SSB | 102 | SARS-CoV-2 Spike S1 RBD |
| 23 | Cockroach | 63 | Liver Cytosol Type 1 (LC1) | 103 | SARS-CoV-2 Spike S1+S2 |
| 24 | Complement C1q | 64 | Liver Kidney Microsome(LKM1) | 104 | SARS-CoV-2 Spike S2 ECD |
| 25 | Core Histone_Citrullinated | 65 | M. Tuberculosis CFP10 | 105 | SARS-CoV-2_S1_RBD(F342L)/40592-V08H6 |
| 26 | Coxsackievirus (B1) Native Antigen | 66 | M. Tuberculosis ESAT-6 Antigen | 106 | Scl-70 |
| 27 | Dengue virus (DENV) | 67 | MDA5 | 107 | Scl-75/Scl-100 (mix) |
| 28 | Diphtheria Toxin | 68 | MERS-CoV NP Protein | 108 | Shrimp_Penacidae |
| 29 | Dog Dander, Mixed-Breed_Canis familiaris | 69 | MERS-CoV Spike Protein (S1 + S2) | 109 | Sm/SmD |
| 30 | Dog Epithelia, Mixed-Breed_Canis familiaris | 70 | Mite, House Dust_Blomia tropicalis | 110 | Streptococcus pneumoniae |
| 31 | dsDNA/ssDNA (mix) | 71 | Mitochondrial Ribosomal Protein S31 / (Human MRPS31) | 111 | TETANUS TOXIN |
| 32 | EBNA1 | 72 | Mumps virus antigen Inactivated | 112 | Thyroglobulin (TG) |
| 33 | Echovirus type 9 | 73 | Mycoplasma Pneumoniae Antigen | 113 | Tissue Transglutaminase (TTG) |
| 34 | Egg-white Lysozyme (HEL) | 74 | Myelin Basic Protein (MBP) | 114 | Toxoplasma Antigen (Toxo) grade II antigen |
| 35 | Endothelial cells antigen | 75 | Myelin oligodendrocytic glucoprotein (MOG) | 115 | U1-sn-RNP (A, BB, C, 68 mix) |
| 36 | Enterovirus 71 VP1 (EV71) | 76 | Myelin-associated Glycoprotein (MAG) | 116 | Varicella Zoster Virus (VZV) Antigen |
| 37 | Fibrinogen IV | 77 | Myosin | 117 | Varicella Zoster Virus (VZV) II Antigen |
| 38 | GBM (kidney) | 78 | Native cytomegalovirus | 118 | Vitronectin |
| 39 | Glucose-6-Phosphatase, Catalytic, 2 /(G6PC2) | 79 | Native Influenza A Virus H3N2 | 119 | West Nile Virus (WNV) Envelope protein |
| 40 | Glutamate decarboxylase 1 (GAD1) | 80 | Native Influenza B Virus Antigen | 120 | Wheat, Whole_Triticum aestivum |
