# Supplementary material for: Virus-Triggered Autoimmunity Was Associated With Hirschsprung's Disease Through Activation of Innate Immunity
Source: J Immunol Res. 2024 Oct 26;2024:4838514. doi: 10.1155/2024/4838514 (PMC11531361; doi:10.1155/2024/4838514)
Supplement: Supporting Information 2 — Table S2. Customized list of autoantibodies. [file 4838514.f2.docx]

**Supplementary table 2.**

| **Customized list of autoantibodies** | | | | | |
| --- | --- | --- | --- | --- | --- |
| **#** | Autoantibody ID | **#** | Autoantibody ID | **#** | Autoantibody ID |
| 1 | 14-3-3 epsilon (RB) | 41 | Gap Junction Protein, gamma 2 (GJC2) | 81 | Neuronal Pentraxin-1/NPTX1 |
| 2 | 14-3-3 protein beta/alpha (YWHAB) | 42 | GAPDH | 82 | NG2/MCSP/CSPG4 |
| 3 | 14-3-3 Protein e/YWHAE (NP) | 43 | Glial fibrillary acidic protein | 83 | Nogo-66 Receptor/Reticulon 4 Receptor/NgR/RTN4R |
| 4 | abCrystallin (CRYAB) (AB) | 44 | Glial Fibrillary Acidic Protein/GFAP | 84 | NOVA2 |
| 5 | abCrystallin (CRYAB) (NP) | 45 | Glutamate dehydrogenase 2 (GLUD2) | 85 | Olfactomedin-4 |
| 6 | ADCY1 | 46 | Glutamate receptor, ionotropic, N-methyl D-aspartate 1 (GRIN1) | 86 | OLIG2 |
| 7 | ADCY2 | 47 | GRIA2 | 87 | Oligodendrocyte specific protein (OSP) |
| 8 | ADCY4 | 48 | GRM1 | 88 | Orphan glutamate receptor delta2?Orphan glutamate receptor delta2? |
| 9 | ADCY5 | 49 | GRP78 | 89 | PCDHA1 |
| 10 | Adult Whole Brain lysates | 50 | heat shock 60kDa protein 1 (chaperonin) (HSPD1) | 90 | PGLYRP1/PGRP-S |
| 11 | AIF-1 (RB) | 51 | Hepatocyte Cell Adhesion Molecule/HepaCAM | 91 | Phophatidylinositol |
| 12 | Allograft Inflammatory Factor 1/AIF1 (NP) | 52 | Homolog 3 (Drosophila) (HOMER3) | 92 | Plastin-2 |
| 13 | Amyloid b-peptide | 53 | Human Astrocyte Lysate | 93 | PNMA2 |
| 14 | Amyloid-like Protein 1/APLP-1 | 54 | Human Myelin P2 Protein/PMP2 | 94 | Profilin 1 |
| 15 | AQP4 (CB) | 55 | Human Neuron Lysate | 95 | Protein kinase C, gamma (PRKCG) |
| 16 | Aquaporin-4 (AQP4) | 56 | ITPR1 | 96 | Receptor Accessory Protein 2 (REEP2) |
| 17 | Brevican Core Protein/BCAN/BEHAB | 57 | Kappa Light Chains | 97 | Recombination Signal Binding Protein For Immunoglobulin kappa J Region (RBPJ) |
| 18 | CAP1 | 58 | KCNJ10 (Avi) | 98 | Resistin |
| 19 | CAP2 | 59 | KCNJ10 (Bio) | 99 | S100 calcium binding protein B (S100B) (Ori) |
| 20 | Caspr2 | 60 | Lactoferrin | 100 | S100 Calcium Binding Protein B (S100B) (NP) |
| 21 | Cell Adhesion Molecule 2 (CADM2) | 61 | Leucine-rich glioma inactivated-2 | 101 | S100-A12 |
| 22 | CHI3L1/YKL40 | 62 | Leucine-Rich Repeat-Containing Protein 2/LRRN2 | 102 | S100A8/A9 Heterodimer |
| 23 | Chitinase-3-Like Protein 1/CHI3L1 | 63 | Lymphocyte Antigen 6H/LY6H | 103 | SLC30A8 / ZNT8 |
| 24 | Collapsing response mediator protein 5 (CRMP5) | 64 | MAG (Ther) | 104 | Solute Carrier Family 17 (SLC17A6) |
| 25 | Contactin-2/CNTN2/TAG-1 | 65 | Metabotropic glutamate receptor 1 | 105 | Sperm associated antigen 16 (SPAG16) |
| 26 | Coronin-1 | 66 | mGluR2 | 106 | Sulfotransferase 4A1/SULT4A1 |
| 27 | CX30/GJB6 | 67 | mGluR3 | 107 | Synapsin I (SYN1), transcript variant Ia |
| 28 | CX43/GJA1 | 68 | mGluR5 | 108 | SYP |
| 29 | Cytokeratin 10 | 69 | Microtubule associated protein 2 | 109 | Transaldolase/TALDO1 |
| 30 | Dipeptidyl Peptidase 6 (DPP6) | 70 | MMP-14 | 110 | Transketolase |
| 31 | Disks Large Homolog 4/DLG4/PSD95 | 71 | MOG | 111 | TSHR |
| 32 | EBNA1 binding protein 2 | 72 | Monosialoganglioside GM2 (NH4+ salt) | 112 | Ubiquitin Carboxyl-Terminal Hydrolase Isozyme L1/UCH-L1 |
| 33 | EDNRA | 73 | Myelin associated glycoprotein (MAG) (Abn) | 113 | Visinin-Like Protein 1/VILIP/VSNL1 |
| 34 | ELAVL2 | 74 | Myelin associated glycoprotein (MAG) (NP) | 114 | ZIC4 |
| 35 | ELAVL3 | 75 | Myelin-associated oligodendrocyte basic protein (MOBP) |  |  |
| 36 | Excitatory Amino Acid Transporter 2 (EAAT2) | 76 | Myeloid cell nuclear differentiation antigen |  |  |
| 37 | GAD1/GAD67 | 77 | Myosin-9 (MYH9) |  |  |
| 38 | Galactocerebroside | 78 | NCAM-1/CD56 |  |  |
| 39 | Gap Junction Protein Beta 1 (GJb1) (CC) | 79 | Neural Cell Adhesion Molecule 1/NCAM-1/CD56 |  |  |
| 40 | Gap Junction Protein Beta 1 (GJB1) (Abn) | 80 | Neurofascin |  |  |
